# Cellular-Level Analysis of Retinal Blood Vessel Walls Based on Phase Gradient Images

**DOI:** 10.3390/diagnostics13223399

**Published:** 2023-11-08

**Authors:** Mircea Mujat, Konstantina Sampani, Ankit H. Patel, Jennifer K. Sun, Nicusor Iftimia

**Affiliations:** 1 Physical Sciences, Inc., 20 New England Business Center, Andover, MA 01810, USA; patel@psicorp.com (A.H.P.); iftimia@psicorp.com (N.I.); 2 Beetham Eye Institute, Joslin Diabetes Center, Boston, MA 02115, USA; konstantina.sampani@joslin.harvard.edu (K.S.); jennifer.sun@joslin.harvard.edu (J.K.S.); 3 Department of Medicine, Harvard Medical School, Boston, MA 02115, USA; 4 Department of Ophthalmology, Harvard Medical School, Boston, MA 02115, USA

**Keywords:** adaptive optics, retinal disease, scanning laser ophthalmoscopy, diabetic retinopathy

## Abstract

Diseases such as diabetes affect the retinal vasculature and the health of the neural retina, leading to vision problems. We describe here an imaging method and analysis procedure that enables characterization of the retinal vessel walls with cellular-level resolution, potentially providing markers for eye diseases. Adaptive optics scanning laser ophthalmoscopy is used with a modified detection scheme to include four simultaneous offset aperture channels. The magnitude of the phase gradient derived from these offset images is used to visualize the structural characteristics of the vessels. The average standard deviation image provides motion contrast and enables segmentation of the vessel lumen. Segmentation of blood vessel walls provides quantitative measures of geometrical characteristics of the vessel walls, including vessel and lumen diameters, wall thickness, and wall-to-lumen ratio. Retinal diseases may affect the structural integrity of the vessel walls, their elasticity, their permeability, and their geometrical characteristics. The ability to measure these changes is valuable for understanding the vascular effects of retinal diseases, monitoring disease progression, and drug testing. In addition, loss of structural integrity of the blood vessel wall may result in microaneurysms, a hallmark lesion of diabetic retinopathy, which may rupture or leak and further create vision impairment. Early identification of such structural abnormalities may open new treatment avenues for disease management and vision preservation. Functional testing of retinal circuitry through high-resolution measurement of vasodilation as a response to controlled light stimulation of the retina (neurovascular coupling) is another application of our method and can provide an unbiased evaluation of one’s vision and enable early detection of retinal diseases and monitoring treatment results.

## 1. Introduction

The retina, the innermost light-sensitive layer of the eye fundus, is a highly metabolically active neurovascular tissue and is vulnerable to diseases that affect its neural or vascular components, thereby potentially resulting in severe vision loss. The retinal endothelium is responsible for the exchange of oxygen, nutrients, and waste. The retina is the tissue with the greatest metabolic demand in the body and since the retinal capillaries lack precapillary sphincters, they remain perfused continuously [1,2]; therefore, retinal oxygen concentration remains high independent of systemic hemodynamics and atmospheric partial pressures [3]. However, even in early diabetes mellitus (DM), there is loss of pericytes and perturbations in retinal vascular physiology. Diabetic retinal microvasculature experiences impair the ability to autoregulate the tone and consequently the blood flow of the retina [4,5,6,7]. Over time, capillary loss and remodeling can occur with late stages of the disease, resulting in the growth of abnormal retinal neovascularization.

Hyperglycemia, inflammation, and hypoxia are key factors of DM that contribute to retinal neurovascular coupling degeneration in eyes with diabetic retinopathy (DR) with a characteristic pericyte loss and alterations in the neuronal cells’ synapses. DR is the most common microvascular complication of DM and the leading cause of blindness among working-age adults in the US [8,9]. It is characterized by a disruption in neurovascular coupling due to alterations in both neural and vascular structures of the retina that accumulate with increased disease severity and precede visual dysfunction. Retinal vascular complications are characteristic features in DR that define DR classification and progression to late-stage DR. Therefore, there is an unmet need for the identification of specific imaging biomarkers for incipient retinal microvascular alterations in early stages of DR that may play a key role in the clinical management of patients and the evaluation of new therapeutic approaches.

The major arteries and veins of the eye are plainly visible by standard color fundus photography. However, the smallest retinal capillaries cannot be fully visualized by using this technique due to the low lateral resolution. One way to reveal more details of the retinal vasculature is the use of fluorescein angiography that requires the injection of a dye into a peripheral vein to enhance contrast. Although this technique can quantify retinal perfusion, it still lacks the resolution for revealing the finest capillaries and it is an invasive method that involves the risk of adverse effects due to the contrast agent. More recently, it was recognized that the motion contrast generated by the flow of erythrocytes through the blood vessels cause inter-frame intensity fluctuations, enabling visualization of the vessels; by plotting the standard deviation of each pixel between video frames, blood flow itself becomes a contrast agent [10]. Images produced in this fashion sometimes compare favorably to fluorescein angiograms [11] but can be difficult to capture reliably due to interference from bright specular reflecting structures like nerve fibers.

Optical coherence tomography angiography (OCTA), recently introduced into clinical practice [12,13,14], provides depth-resolved imaging of the retinal vasculature. Different implementations detect the motion of the blood by measuring the variation in amplitude or phase of the OCT signal between consecutive axial profiles or cross-sectional scans [15,16]. However, capillaries or lesions such as microaneurysms (MAs) and neovascularization with slow flow can be missed or partially visualized and the wall morphology cannot be analyzed with OCT.

A new approach for imaging smaller arterioles and venules involves adaptive optics (AO) to increase image resolution and quality. Ocular aberrations due to imperfections in the cornea, lens, and tear film are measured with a wavefront sensor and are corrected by a wavefront compensator in a closed-loop control algorithm. Real-time measurement of ocular aberrations is generally performed using a Hartmann–Shack wavefront sensor comprising a lenslet array and a CCD camera [17], and the wavefront correction is performed with deformable mirrors [18] or liquid crystal phase modulators [19,20].

Scanning laser ophthalmoscopes (SLOs) have been used for a long time to image the retina in a confocal configuration using a pinhole in front of the detector that enables light collection only from the retinal location conjugate to the pinhole. However, the eye can only produce a diffraction-limited illumination spot on the retina for a small pupil diameter less than ~2 mm, and SLOs can achieve a lateral resolution of approximately 8–10 µm. To increase the lateral resolution of the retinal image, the eye pupil needs to be dilated such that a larger diameter imaging beam (6–9 mm) can be used to enable a tighter focus on the retina. As the pupil diameter is increased, ocular aberrations dynamically deform the imaging beam and AO is needed to correct ocular aberrations in real time; therefore, AO-corrected SLOs have been developed (AO-SLOs) to produce diffraction limited illumination spots (~2.5 µm) on the retina [21] for the best possible imaging performance.

Nevertheless, due to a low refractive index contrast, the capillaries are relatively transparent and difficult to visualize even in high-resolution imaging of the retina as provided by AO-SLOs in a typical reflective confocal configuration. Only dim specular reflections from blood cells or the shadow cast on deeper layers can be seen sometimes. About a decade ago, a new method was introduced to take advantage of the multiply scattered light (as opposed to direct backscatter in a reflective confocal SLO) and to enable visualization of the retinal capillaries with unprecedented details [22]. By using a larger, offset pinhole in front of the detector, the direct back-reflected light is rejected and forward-scattered light reflected by deeper layers is collected. In this way, mostly forward-scattering structures such as erythrocytes and vessel walls became dominant features and can be visualized. This method, sometimes referred to as “dark field imaging” has lower contrast due to the collection of multiple scattered light; however, the diffraction-limited illumination spot provided by AO preserves the high-resolution advantage of AO-SLOs. The foremost benefit of this method is that it enables the visualization of retinal microstructures such as the blood vessel walls, blood cells, nerve bundles, or the cones outer segment that cannot be seen in confocal configurations. Large particles in Mie scattering regime and phase objects scatter mostly forward, and there is a lot of information in these forward-scattered photons. Such non-confocal AO-SLO detection methods reveal additional details of the microvascular structure not seen before and enable significant progress in retinal vascular imaging [23].

## 2. Materials and Methods

### 2.1. The Imaging System

**Figure 1:**
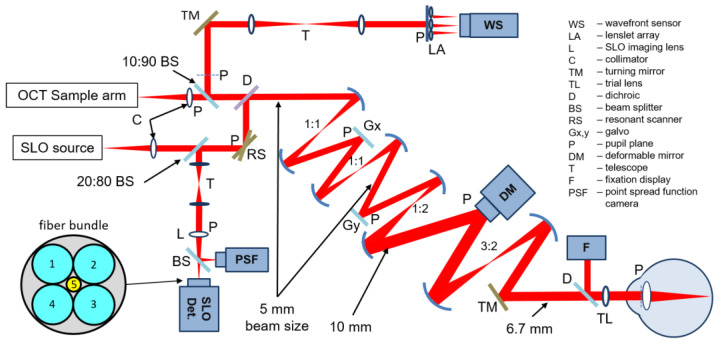
Optical diagram of MAORI.

We developed a multimodal AO retinal imager (MAORI) as a modular, compact, clinical prototype that allows researchers, in the clinic or the lab, to explore the fine cellular and lamellar retinal structure of subjects’ retinas with near-isotropic micron-level resolution. The multimodal system includes a scanning laser ophthalmoscope (SLO) with closed-loop AO wavefront sensing and compensation, and a spectral domain optical coherence tomography (SDOCT) channel. The OCT channel provides complimentary cross-sectional images (B-scans) simultaneously recorded and co-registered with the SLO image. The OCT beam is also the wavefront sensor (WS) beacon. A fixation display and a point spread function camera are also included, as shown in the optical diagram in Figure 1. To make the system more user-friendly, several components were motorized to position the patient interface, to focus the fixation display, to adjust the length of the OCT delay line, and to tune the polarization controller.

Following the demonstration of offset aperture AO-SLO, a new technique, split-detector imaging [24], has been proposed to visualize the outer segment of cone photoreceptors. This technique adopted to retinal imaging was originally introduced in differential phase-contrast microscopy [25]. Two offset apertures optically conjugated to opposite sides of the illumination spot on the retina are used simultaneously. The split-detector image is calculated as the difference of the two offset images divided by their sum. The confocal image is recorded through a confocal aperture simultaneously with the split-detector image, and therefore, the two images are perfectly co-registered spatially and temporally. This method works well when imaging disk-like structures, such as red blood cells and photoreceptors, and is very sensitive to edges perpendicular to the split direction; however, it is less sensitive to discontinuities along the direction of the two offsets. To visualize the wall boundaries of blood vessels oriented parallel to the split direction, the imager operator needs to reconfigure the position of the offset apertures and that is not practical in a clinical environment. Another technique demonstrated in 2017 [26] involves imaging the same retinal location by repositioning the offset aperture sequentially at different distances and angles with respect to the illumination spot. Exquisite details of retinal structures not seen before can be revealed with this “multi-offset” approach; however, the sequential acquisition of offset images makes it very tedious, time-consuming, and difficult to use in the clinic.

A new detection scheme has been introduced recently [27] with an arrangement of light-collecting fibers that removes the disadvantages mentioned above and provides isotropic imaging while retaining all the advantages of offset aperture and split-detector imaging. The detection scheme consists of four optical fibers (1–4) arranged as a compact bundle, as shown in the bottom-left side of Figure 1. The two orthogonal pairs provide the split-detector imaging in orthogonal directions, simultaneously removing the directionality disadvantage of the one-offset or one-split configurations. Another fiber in the center of the bundle (5) provides the confocal image. The core diameter of the central fiber is 50–100 µm, and the core diameter of the offset fibers is 600 µm. The fibers are glued inside the ferule of a fiber optic connector and are polished to provide an optical quality surface. Standard fiber connectors are used at the other end of the fibers to attach them to APD detectors, and all five images are acquired simultaneously through a multi-channel digitizer. Any AO-SLO system can be easily retrofitted to use this simple imaging configuration. The imaging system based on this detection arrangement has been used for imaging the structure of blood vessels, and vascular and neural retinal networks. As a similar concept, Austin Roorda’s group presented the use of a bundle with seven fibers, initially developed for multiple sampling and pixel reassignment within the Airy disk to enhance the signal-to-noise ratio (SNR) [28]. They also showed that by flipping the telescope in front of the focal plane, one can collect the confocal photons with the center fiber and the multiple scattered photons with the exterior fibers; however, because the diameter of the central fiber is large, a special mask is needed to create the confocal aperture. Following their original demonstration of split detection, Alfredo Dubra’s group presented a free-space optics arrangement [29] in which they added at second split orthogonal to the first one, now reading simultaneously four offset apertures and the confocal. As opposed to the fiber bundle configuration, the free-space arrangements are difficult to align and maintain.

### 2.2. Human Subjects and Imaging Procedure

This was an IRB-approved observational study conducted at the Beetham Eye Institute of the Joslin Diabetes Center in Boston, evaluating adults with type 1 diabetes (T1D). The exclusion criteria included pupillary miosis or an inability to dilate, prior panretinal photocoagulation, non-diabetic retinal pathology, media opacities limiting the ability to acquire high-quality AOSLO/OCT images, and hypertension. Each study eye underwent mydriasis, ultrawide fundus photography (UWF), and AOSLO/OCT imaging in a single-visit study. DR severity was graded by certified graders on colored UWF based on the ETDRS classification system.

### 2.3. Confocal and Non-Confocal Imaging

**Figure 2:**
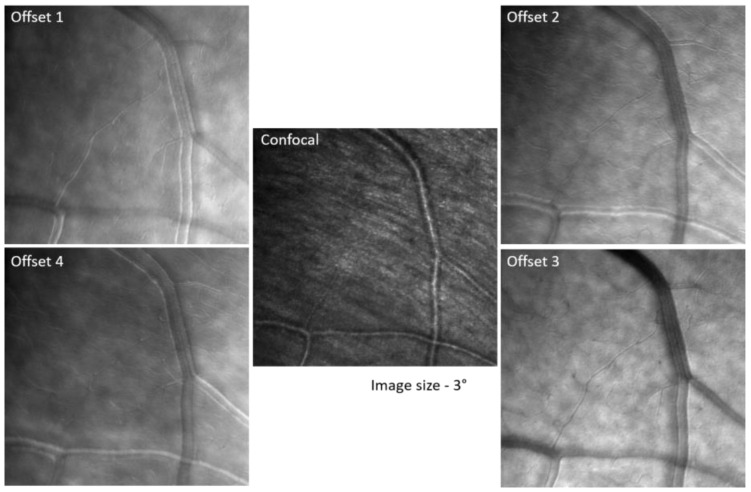
Offset and confocal images obtained with the 5-fiber bundle.

An example of images obtained with the 5-fiber bundle including the confocal and the four offset channels is shown in Figure 2. It is important to notice here the complementarity of these images acquired simultaneously: the confocal mode provides a clear image of the nerve fiber bundles in the retinal nerve fiber layer (RNFL) but no details of the small capillaries or the wall of large vessels, while the offset mode shows no RNFL fiber bundles but the capillaries and the vessel wall details pop up clearly.

**Figure 3:**
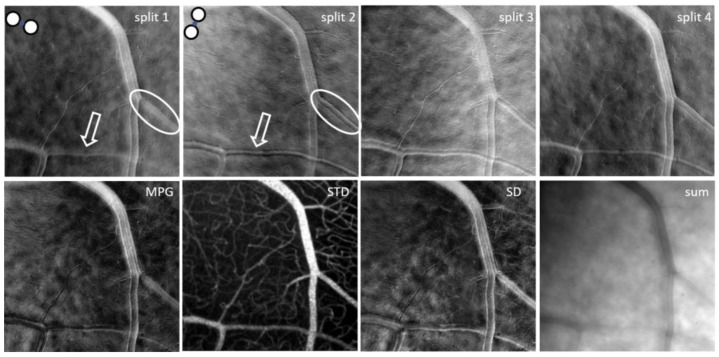
Mean images for the four splits, magnitude of the phase gradient (MPG), mean of the four split standard deviations (STD), standard deviation among the four simultaneous offsets (SD), and sum derived from the four offset images shown in Figure 2.

A significant problem with split detection is its directionality artefact. If a blood vessel is parallel with the offset/split direction, it cannot be seen, as illustrated in the split-1 image within the white oval in Figure 3. However, the vessel edges can be distinguished very well if the offset/split direction is perpendicular to the vessel, as within the white oval in the split-2 image of Figure 3. Similarly, the white arrows point to crisp wall details of a blood vessel in the split-2 image and to blurred details in the split-1 image of Figure 3. It should therefore be expected that if only one split is used, there will be structures that cannot be visualized and one needs two orthogonal split directions simultaneously for complete visualization of all retinal structures, independent of their orientation.

Typically, a stack of 100–200 images is recorded at one location. The images are aligned using a non-rigid registration method [30], and one advantage of recording multiple channels simultaneously is that all of them can be aligned with only one alignment function. Mean and standard deviation images are obtained. The mean image cleans up the speckle and enhances the details of microscopic structures. The standard deviation image shows motion contrast. The motion of blood through capillaries generates large standard deviation values, while places where nothing moves exhibit low standard deviation values; this concept is similar to motion contrast employed in OCTA. This analysis can be applied to each individual offset channel.

Split-detection analysis can be performed using multiple combinations of the four offset images. Two orthogonal split images are generated for fiber pairs 1–3 (split-1) and 2–4 (split-2) as illustrated in Figure 3. Two additional split images are obtained by first adding adjacent fibers, for example 1 + 2 and 3 + 4, and then performing subtraction divided by the sum of the two sums (1 + 2 − 3 − 4)/(1 + 2 + 3 + 4) for a near vertical split (split-4); similarly, (1 − 2 − 3 + 4)/(1 + 2 + 3 + 4) provides an near horizontal split (split-3). Therefore, four split images can be obtained; for the horizontal, vertical, and diagonal (±45 deg) directions of the offset apertures and given their directionality, each of the four split images highlights boundaries such as blood vessel walls along different directions. In addition to this, we can calculate the mean and standard deviation for each split similar to individual offsets and the mean of the four split standard deviations (STD).

The horizontal and vertical splits are similar to the half aperture, as in [24]. They can also be interpreted as phase derivatives along the horizontal and vertical directions. As mentioned above, the idea of split detection was derived from differential phase-contrast microscopy; therefore, using these orthogonal derivatives, one can directly calculate the magnitude of the phase gradient (MPG) as the square root of the sum of squared orthogonal derivatives or reconstruct the phase [31,32,33].

The standard deviation (SD) among the four simultaneous offsets, a concept first introduced in [26], highlights the differences illustrated in these images, which are mostly visible at the blood vessel boundaries, due to the directionality diversity provided by such a multi-offset detection arrangement. The sum of the four offset images in a ring-detection configuration (sum) combines all detected forward-scattered light while rejecting the direct backscattered light, which is simultaneously detected through the central fiber in the confocal image (SLO). Movies for monitoring temporally dynamic processes such as blood flow or cell migration, as well as mean and standard deviation images for each of the four splits, SD, sum, STD, and MPG, can be generated through our analysis. An example of all these mean images is shown in Figure 3. Also as an example, Video S1 (Supplementary Materials) shows the stack of split-4 images that could be registered and aligned after removing the images distorted by involuntary eye motion. The video illustrates the flow of blood cells through the capillaries that generates the motion contrast highlighted in the STD image in Figure 3.

It should be noted here that SD, STD, sum, and MPG are all isotropic, direction-independent, as they were derived from all four offset images and are therefore directionally diverse. STD reveals maps of capillaries with much better contrast than those in other imaging modalities and in a mode similar to OCTA. STD can also be used to segment the flow location in large vessels and to quantify the inside diameter of the vessels.

## 3. Results

**Figure 4:**
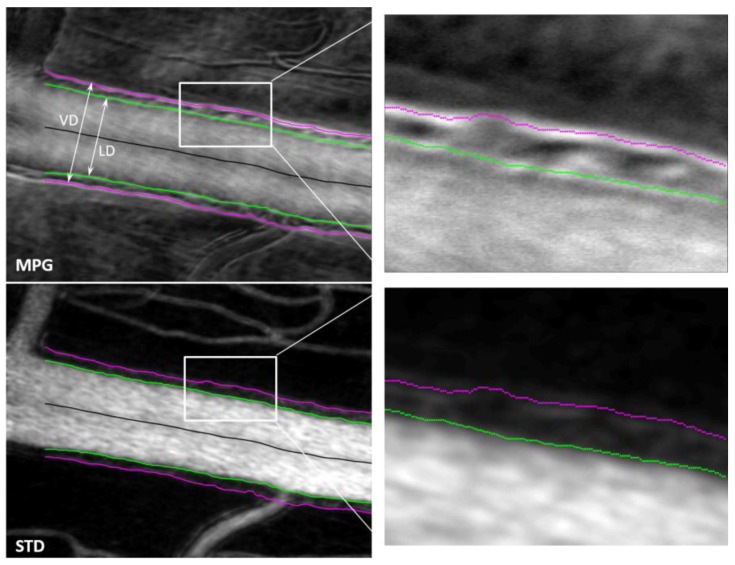
MPG (**top**) and STD (**bottom**) images with segmented vessel walls (magenta and green). The right-side column shows a magnified view of the white rectangle area in the left-side images. (VD—vessel diameter; LD—lumen diameter).

Two orthogonal split-detector images are used to calculate the magnitude of the phase gradient, which highlights local variations in the refractive index. The gradient maxima indicate the location of structural changes (edges) associated with the blood vessel wall boundaries. STD images highlight the blood flow through motion contrast, and the edges of the flow shown in these images correspond to the lumen boundaries. Segmentation of the gradient maxima and of the STD images provides the inside and outside boundaries of the vessel walls, as illustrated in Figure 4, and enables calculation of the wall-to-lumen ratio (WLR) and of other structural characteristics of the retinal vasculature.

**Figure 5:**
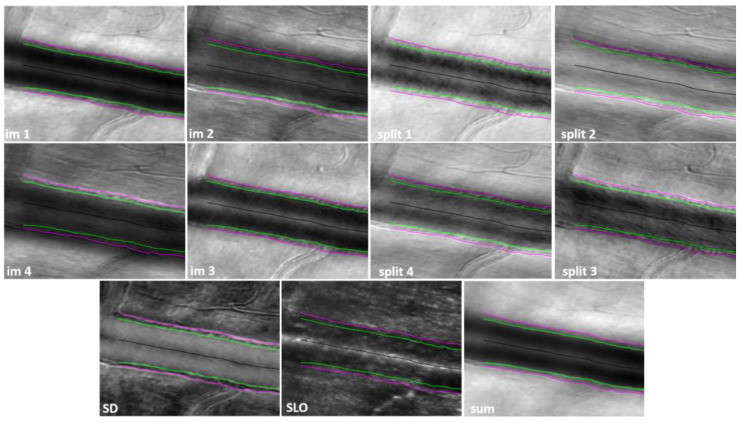
Offset (im 1–4), split (1–4), SD, SLO, and sum images with segmented vessel walls (VD—magenta and LD—green).

**Figure 6:**
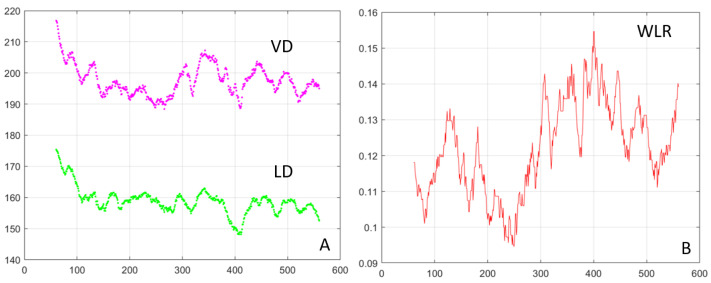
(**A**) Vessel diameter (VD—magenta) and lumen diameter (LD—green) for the scan shown in Figure 5; axes in µm. (**B**) Wall-to-lumen ratio.

An example of the measured images (offset images im 1–4 and SLO) and derived images (split 1–4, SD, sum) is shown in Figure 5 with the blood vessel boundaries segmented and highlighted with magenta (vessel boundary on the outside) and green (lumen boundary on the inside). Figure 6A shows the vessel (VD—magenta) and the lumen (LD—green) diameters as a function of the position along the vessel. WLR is calculated as $WLR=\frac{0.5*\left(VD-LD\right)}{LD}$ and is shown in Figure 6B. VD and LD are measured in µm; WLR is adimensional; and the horizontal axis in both plots represents the position along the vessel in µm.

**Figure 7:**
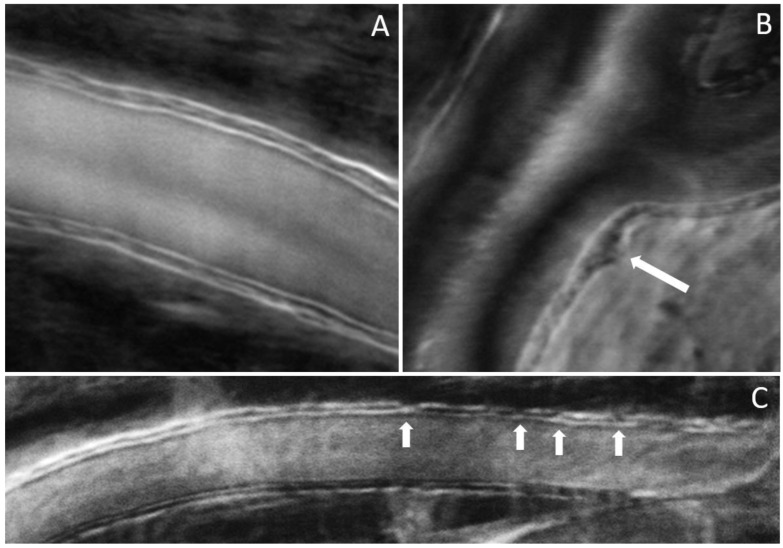
(**A**) Control; (**B**,**C**) no DR; arrows indicate disruption of the wall integrity.

**Figure 8:**
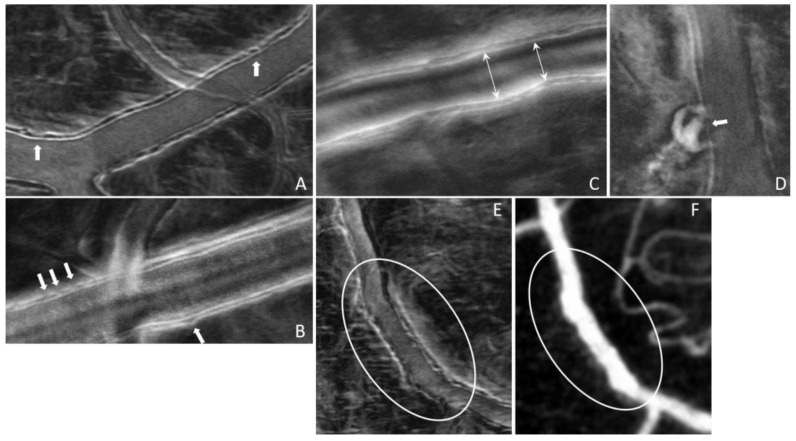
(**A**,**B**) Mild NPDR; (**C**) moderate NPDR; (**D**) severe NPDR; (**E**,**F**) PDR; arrows in (**A**,**B**) indicate disruption of the wall integrity; arrow in (**D**) indicates a microaneurysm.

**Figure 9:**
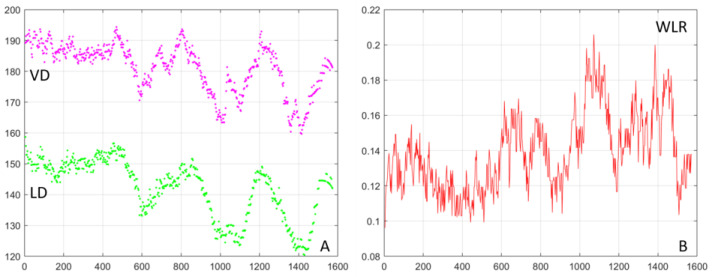
(**A**) Vessel diameter (VD—magenta) and lumen diameter (LD—green) for a scan partially shown in Figure 8C; axes in µm. (**B**) Wall-to-lumen ratio (WLR).

The results shown in Figure 4, Figure 5 and Figure 6 were obtained from a healthy subject. Figure 7, Figure 8 and Figure 9 show images obtained from eyes with different levels of DR severity. Figure 7A is from the same control as in Figure 4, Figure 5 and Figure 6. Figure 7B,C are from two subjects with T1D but no DR. Figure 8A,B correspond to subjects with mild nonproliferative DR (NPDR), C is from a moderate NPDR subject, D is from a severe NPDR subject, and E/F is from a proliferative DR (PDR) subject. All images in Figure 7 and Figure 8 are MPG except for Figure 8F, which is the STD image corresponding to Figure 8E. Similarly to Figure 6, Figure 9A shows the vessel diameter (VD—magenta) and lumen diameter (LD—green) as a function of position along the vessel for a scan partially shown in Figure 8C, and Figure 9B displays the WLR.

## 4. Discussion

Diffraction-limited imaging, as in AO-SLO, is generally performed with a large diameter illumination beam (6.7 mm in our case, as shown in Figure 1) that enables a diffraction-limited illumination spot on the retina with an approximately 2.5–3 µm diameter (for a normal eye) given active compensation of the eye aberrations through AO. The spot diameter depends on the eye length and varies from subject to subject. In the offset channels, the image resolution is dictated by the size of the illumination spot as the technique is a flying-spot. In the confocal channel, an additional pinhole in front of the detector limits detection of light only from the illumination spot and additionally rejects out-of-plane photons enhancing the contrast of the confocal image. The lower contrast in the offset channels is due to the collection of multiply scattered photons; however, the resolution is the same in the confocal and offset images, as provided by the size of the illumination spot.

Various retinal structures have a different refractive index than the tissue surrounding them. Minute variations with respect to the surroundings generate little contrast and render the structures basically transparent in transmission or hard to see in reflection. Phase imaging is sensitive to such small refractive index variations and provides additional contrast to image such structures, sometimes called phase objects. Phase gradients can be used to identify the borders of phase objects, which represent the location where the refractive index has the largest jump. Quad imaging, as presented here with two orthogonal split detection directions, can be used to calculate MPG, which highlights local variations in the refractive index. The gradient maxima indicate the location of the structural changes (edges) associated with the blood vessel wall boundaries, as shown in Figure 4. STD images highlight the blood flow through motion contrast and the edges of the flow shown in these images correspond to the lumen boundaries. Segmentation of the gradient maxima and of the STD images provides the inside (green in Figure 4 and Figure 5) and outside (magenta) boundaries of the vessel walls and enables the calculation of VD, LD, WLR, and of other structural characteristics of the retinal vasculature that have diagnostic value.

Segmentation of the MPG and STD images is currently performed semi-automatically using a custom-made Matlab program that identifies the gradient maxima in the MPG image and the borders of the flow in the STD image. Some gradient maxima such as the borders of the mural cells within the vessel wall, as can be seen in the top right image in Figure 4, are then removed manually by an expert grader (MM). Discontinuities of the identified wall boundaries are also corrected manually. The inside border of the wall is segmented simultaneously in the MPG and STD images, and the right column in Figure 4 shows a good correlation between the edge of the flow in the STD image with the gradient maxima in the MPG image, both in green. The outside border of the wall can only be segmented in the MPG image since there is no blood flow at that location to create motion contrast.

The MPG and STD images were chosen for wall segmentation because they are isotropic, independent of the offset/split direction since they were derived from all four offset/split images. SD, SLO, and sum are also isotropic, as illustrated in Figure 5. However, the wall boundaries are not visible at all in the confocal/SLO image, sum has low contrast at the borders, and SD is very similar to MPG. In addition, individual offsets and splits are not useful for vessel segmentation since the appearance of the vessel wall varies with the orientation of the vessel with respect to the image. One side of the vessel might show white boundaries, while the other side might show dark boundaries and border identification becomes very difficult. If the vessel is oriented along the offset/split direction, the boundary is lost, as illustrated in Figure 3, and cannot be segmented. MPG and STD images are the most reliable for vessel segmentation.

The imaging method and the analysis procedure presented here enable the identification of structural abnormalities that are the result of disease, DR due to T1D in particular in this study. Loss of structural integrity of the blood vessel wall may result in MAs, a hallmark lesion of DR, which may leak or rupture and further cause visual decline. Figure 7 and Figure 8 show several examples of structural distortions in subjects with various levels of DR severity. The healthy eye illustrated in Figure 7A has strong continuous vessel borders. In contrast, the rest of the images in Figure 7 and Figure 8 show disruptions in the vessel walls, indicated by the arrows, either bulging or local thinning of walls that may become the location of future MAs like the one shown in Figure 8D (arrow). Additional videos (Videos S2 and S3) and an image (Figure S1) are included in the Supplementary Materials to illustrate very faint blood flow through the MA, which is confirmed in the STD image by the very dim motion contrast bulge next to the blood vessel. Figure 8E,F show significant variations in the lumen diameter in a portion of the vessel that has no additional branches (white oval) for a PDR subject. It is expected that the lumen diameter changes across a branch for flow conservation while it should be relatively constant in between branches. DR might affect the elasticity/stiffness of the vessel wall, and distortions could occur, such as those illustrated here. Similarly, Figure 9 shows tremendous variations in the lumen and vessel diameters along a vessel of an eye with moderate NPDR. It seems that the wall thickness is relatively constant while the lumen/vessel diameter varies significantly (also arrows in Figure 8C). The result is a large variation in WLR along the vessel, as shown in Figure 9B, in between branches. Future prospective studies may elucidate whether increased variation in WLR is associated with future problems related to vessel wall integrity.

## 5. Conclusions

The AO imaging modality described here enables the visualization and characterization of retinal blood vessels with cellular resolution. The segmentation of blood vessel walls provides quantitative measures of geometrical characteristics of the vessel walls, including vessel and lumen diameters, wall thickness, and wall-to-lumen ratio. Retinal diseases such as DR affect the structural integrity of the vessel walls, their elasticity, their permeability, and their geometrical characteristics. The ability to measure disease-induced changes may prove valuable for understanding the vascular effects of retinal diseases, monitoring disease progression, and drug testing. A study relating WLR changes to the level of DR severity is ongoing. Early detection of such abnormal alterations may also open new treatment avenues for vision preservation.

Functional testing of retinal circuitry can provide an unbiased evaluation of visual function, enable early detection of retinal diseases, and allow for monitoring of the response to treatment. Light stimulation evokes neuronal activity in the retina, resulting in the dilation of retinal blood vessels and increased blood flow. This response, named functional hyperemia or neurovascular coupling, brings additional oxygen and nutrients to active neurons and is one of the critical physiological mechanisms that allow for the adaptation of the retinal vascular system to changing neural function and increased metabolic demand. The analysis method described here enables future high-resolution measurement of vasodilation as a response to controlled light stimulation of the retina and may reveal functional alterations in diseased eyes that could warrant early interventions.

## Data Availability

Data can be made available upon request subject to PSI approval.

## Supplementary Materials

The following supporting information can be downloaded at https://www.mdpi.com/article/10.3390/diagnostics13223399/s1, Video S1: Split-4 video of the scan shown in Figure 3, Video S2: confocal video of the MA shown in Figure 8D, Video S3: Split-4 video of the MA shown in Figure 8D, Figure S1: STD image of the video of the MA shown in Figure 8D.

## References

[1] Fulton A.B., Akula J.D., Mocko J.A., Hansen R.M., Benador I.Y., Beck S.C., Fahl E., Seeliger M.W., Moskowitz A., Harris M.E. (2009). Retinal degenerative and hypoxic ischemic disease. Doc. Ophthalmol..

[2] Steinberg R.H. (1987). Monitoring communications between photoreceptors and pigment epithelial cells: Effects of “mild” systemic hypoxia. Friedenwald lecture. Investig. Ophthalmol. Vis. Sci..

[3] Shakib M., De Oliveira L.F., Henkind P. (1968). Development of Retinal Vessels. II. Earliest Stages of Vessel Formation. Investig. Ophthalmol. Vis. Sci..

[4] Bek T., Hajari J., Jeppesen P. (2008). Interaction between flicker-induced vasodilatation and pressure autoregulation in early retinopathy of Type 2 diabetes. Graefe’s Arch. Clin. Exp. Ophthalmol..

[5] Bower B., Zhao M., Zawadzki R., Izatt J. (2007). Real-time spectral domain Doppler optical coherence tomography and investigation of human retinal vessel autoregulation. J. Biomed. Opt..

[6] Tilma K.K., Bek T. (2012). Topical treatment for 1 week with latanoprost but not diclofenac reduces the diameter of dilated retinal arterioles in patients with type 1 diabetes mellitus and mild retinopathy. Acta Ophthalmol..

[7] Ye X.D., Laties A.M., Stone R.A. (1990). Peptidergic innervation of the retinal vasculature and optic nerve head. Investig. Ophthalmol. Vis. Sci..

[8] Ciulla T.A., Amador A.G., Zinman B. (2003). Diabetic Retinopathy and Diabetic Macular Edema: Pathophysiology, screening, and novel therapies. Diabetes Care.

[9] (2021). IDF Diabetes Atlas Tenth Edition. https://diabetesatlas.org/.

[10] Tam J., Martin J.A., Roorda A. (2010). Noninvasive visualization and analysis of parafoveal capillaries in humans. Investig. Ophthalmol. Vis. Sci..

[11] Chui T.Y., Zhong Z., Song H., Burns S.A. (2012). Foveal avascular zone and its relationship to foveal pit shape. Optom. Vis. Sci..

[12] Spaide R.F., Fujimoto J.G., Waheed N.K., Sadda S.R., Staurenghi G. (2017). Optical coherence tomography angiography. Prog. Retin. Eye Res..

[13] Kashani A.H., Chen C.L., Gahm J.K., Zheng F., Richter G.M., Rosenfeld P.J., Shi Y., Wang R.K. (2017). Optical coherence tomography angiography: A comprehensive review of current methods and clinical applications. Prog. Retin. Eye Res..

[14] Jia Y., Bailey S.T., Hwang T.S., McClintic S.M., Gao S.S., Pennesi M.E., Flaxel C.J., Lauer A.K., Wilson D.J., Hornegger J. (2015). Quantitative optical coherence tomography angiography of vascular abnormalities in the living human eye. Proc. Natl. Acad. Sci. USA.

[15] Fingler J., Zawadzki R.J., Werner J.S., Schwartz D., Fraser S.E. (2009). Volumetric microvascular imaging of human retina using optical coherence tomography with a novel motion contrast technique. Opt. Express.

[16] Makita S., Hong Y., Yamanari M., Yatagai T., Yasuno Y. (2006). Optical coherence angiography. Opt. Express.

[17] Prieto P.M., Vargas-Martin F., Goelz S., Artal P. (2000). Analysis of the performance of the Hartmann-Shack sensor in the human eye. J. Opt. Soc. Am. A-Opt. Image Sci. Vis..

[18] Doble N., Yoon G., Chen L., Bierden P., Singer B., Olivier S., Williams D.R. (2002). Use of a microelectromechanical mirror for adaptive optics in the human eye. Opt. Lett..

[19] Shirai T. (2002). Liquid-crystal adaptive optics based on feedback interferometry for high-resolution retinal imaging. Appl. Opt..

[20] Vargas-Martin F., Prieto P.M., Artal P. (1998). Correction of the aberrations in the human eye with a liquid-crystal spatial light modulator: Limits to performance. J. Opt. Soc. Am. A-Opt. Image Sci. Vis..

[21] Romero-Borja F., Venkateswaran K., Roorda A., Hebert T. (2005). Optical slicing of human retinal tissue in vivo with the adaptive optics scanning laser ophthalmoscope. Appl. Opt..

[22] Chui T.Y., Vannasdale D.A., Burns S.A. (2012). The use of forward scatter to improve retinal vascular imaging with an adaptive optics scanning laser ophthalmoscope. Biomed. Opt. Express.

[23] Roorda A., Duncan J.L. (2015). Adaptive Optics Ophthalmoscopy. Annu. Rev. Vis. Sci..

[24] Scoles D., Sulai Y.N., Langlo C.S., Fishman G.A., Curcio C.A., Carroll J., Dubra A. (2014). In Vivo Imaging of Human Cone Photoreceptor Inner Segments. Investig. Ophthalmol. Vis. Sci..

[25] Hamilton D.K., Sheppard C.J.R. (1984). Differential phase contrast in scanning optical microscopy. J. Microsc..

[26] Rossi E.A., Granger C.E., Sharma R., Yang Q., Saito K., Schwarz C., Walters S., Nozato K., Zhang J., Kawakami T. (2017). Imaging individual neurons in the retinal ganglion cell layer of the living eye. Proc. Natl. Acad. Sci. USA.

[27] Mujat M., Patel A., Maguluri G., Ferguson R.D., Iftimia N. (2021). Simultaneous Multi-Offset Imaging of Retinal Microstructures Free of Directionality Artifacts.

[28] Mozaffari S., Jaedicke V., Larocca F., Tiruveedhula P., Roorda A. (2018). Versatile multi-detector scheme for adaptive optics scanning laser ophthalmoscopy. Biomed. Opt. Express.

[29] Sredar N., Kowalski B., Razeen M.M., Steven S., Dubra A. (2018). Non-confocal quad-detection adaptive optics scanning light ophthalmoscopy of the photoreceptor mosaic. Investig. Ophthalmol. Vis. Sci..

[30] Mujat M., Akula J.D., Fulton A.B., Ferguson R.D., Iftimia N. (2023). Non-rigid registration for high-resolution retinal imaging. Diagnostics.

[31] Bronstein R., Werman M., Peleg S. (1992). Surface reconstruction from derivatives. Proceedings of the International Conference on Pattern Recognition.

[32] Dubra A., Paterson C., Dainty C. (2004). Wave-front reconstruction from shear phase maps by use of the discrete Fourier transform. Appl. Opt..

[33] Freischlad K.R., Koliopoulos C.L. (1986). Modal estimation of a wave front from difference measurements using the discrete Fourier transform. J. Opt. Soc. Am. A.

