# Supplementary figures and images for: Cellular-Level Analysis of Retinal Blood Vessel Walls Based on Phase Gradient Images
Source: Diagnostics (Basel). 2023 Nov 8;13(22):3399. doi: 10.3390/diagnostics13223399 (PMC10670340; doi:10.3390/diagnostics13223399)

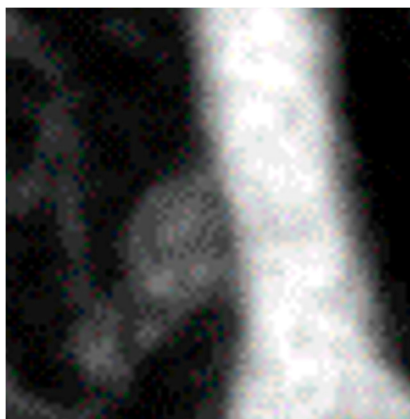

Figure S1: STD image of the video of the MA shown in Figure 8D.

Supplement: Supplementary file 1 [file diagnostics-13-03399-s001.zip › Figure S1.pdf]
